# Influence of fabrication method on the marginal fit of temporary restorations

**DOI:** 10.1590/1807-3107bor-2024.vol38.0063

**Published:** 2024-07-15

**Authors:** Thaís Cristina Mendes RODRIGUES, Caio Cesar Dias RESENDE, Guilherme Faria MOURA, Fábio Henrique de Paulo Costa SANTOS, Gustavo MENDONÇA, Karla ZANCOPE, Flávio Domingues NEVES

**Affiliations:** (a) Private practice. Uberlândia, MG, Brazil.; (b) Centro Universitário do Triângulo – Unitri, Department of Implantology, Uberlândia, MG, Brazil.; (c) Medical University of South Carolina, Department of Reconstructive and Rehabilitation Science, Charleston, SC, USA.; (d) Universidade Federal de Uberlândia – UFU, School of Dentistry, Department of Occlusion, Fixed Prosthesis and Dental Materials, Uberlândia, MG, Brazil.; (e) Virginia Commonwealth University, School of Dentistry, Department of General Practice, Richmond, VA, USA.

**Keywords:** Tooth Crown, Printing, Three-Dimensional, Dental Marginal Adaptation

## Abstract

Computer-aided manufacturing (CAM) technology allows the use of different manufacturing techniques. This in vitro study aimed to evaluate the marginal fit of temporary restorations manufactured using conventional chairside methods, milling, and three-dimensional printing. Fifteen 3-element temporary restorations specimens were produced and categorized into three groups: non-digital, obtained using the conventional chairside method (GC); milled (GM); and three-dimensionally printed (GP). Marginal fit was assessed using scanning electron microscopy (SEM) performed under two conditions: one with only the central screw tightened, and the other with all three screws tightened. Horizontal misfit values were categorized as over-, equal-, and under-extended and qualitatively analyzed. Statistical analysis was performed using the Tukey–Kramer test (α=0.05). In the vertical assessment, three-dimensionally printed restorations demonstrated greater misfit than restorations obtained by milling and the conventional chairside method (P<0.05). In the horizontal assessment, the misfit in the GP group was significantly higher than that in the GM and GC groups. Restorations obtained using the conventional chairside method and milled provisional restorations showed more favorable results than three-dimensionally printed restorations.

## Introduction

The rapid production of temporary restorations using milling machines or three-dimensional printers with computer-aided manufacturing (CAM)^1-3^ is one of the benefits of digital technology. The importance of temporary restorations in oral rehabilitation is unquestionable considering their role in gingival conditioning and the success of the final restoration.^4^ A digital workflow can also reduce the number of clinical steps and make the clinical outcomes more independent of the operator’s technical skills.^5,6^ Milled prosthetic restorations have shown acceptable levels of marginal fit^7^ while achieving satisfactory resistance and aesthetics,^8^ and have gained greater acceptance with digital workflows in dental clinical protocols.^9^

Three-dimensional printing is an additive manufacturing technique used in digital flow. The American Society for Testing and Materials (ASTM) defines additive manufacturing (AM) as “a process of joining materials to make objects from three-dimensional cast data, usually layer upon layer, as opposed to subtractive manufacturing methodologies.”^10,11^ This manufacturing technique can produce less waste, ensure cost savings, reduce the need for storage of raw materials, and minimize the environmental impact of the procedure.^12-15^ This approach can also reduce the time of intraoral exposure and the number of appointments to the dental office.^16-19^

Passive seating and marginal fit of prosthetic structures are desirable characteristics of prosthetic restorations.^20-22^ These are indispensable for balancing the mechanical and biological aspects of restorations, and help reduce the load on the prosthetic abutment, screw, and surrounding bone.^23-25^ The absence of these features could cause several problems of biological origin, including bacterial infiltration, peri-implantitis, pain, and inflammation with bone loss,^26^ loosening and fracture of screws, risk of fracture of the prosthetic component, and even loss of osseointegration.^26^ This passivity could be measured using the Sheffield Test.^25^ Scanning electron microscopy (SEM) is used for analyzing and measuring restoration misfit.^27^

A retrospective study^27^ that evaluated the success of single immediate implant rehabilitation (514 implants placed in 332 patients) showed a biomechanical complication rate of the provisional prosthesis of 9.6% (57 rehabilitations in 38 patients). Furthermore, a prospective cohort study^29^ that evaluated the clinical outcomes of 215 single-immediate supported implant rehabilitations in 215 patients showed complication rates of 15% for provisional restorations, mainly due to fracture or screw loosening. These fractures indicate the necessity of unscrewing the provisional abutment, which can induce biological complications (that is periimplantar bone loss).^30^ Indeed, these previous findings indicate the necessity of improving provisional rehabilitation supported by dental implants. Therefore, this study aimed to evaluate the marginal fit of temporary restorations manufactured using the conventional chairside method, milling, and three-dimensional printing. The null hypothesis was that provisional restorations manufactured using these three techniques would not differ.

## Methods

The present study followed a 1 × 3 factorial design with marginal fit as the main study factor for the comparison of three methods of manufacturing provisional restorations: the conventional chairside method (GC), milling (GM), and three-dimensional printing (GP). The misfit of the implant structures in each cast was evaluated using SEM (SEM VEGA; Tescan, Brun, Czech Republic). The number of specimens (n = 5) was determined by power analysis based on previous studies from the research group. For this, a target power of 0.8, α = 0.05 and a minimum difference of 25 microns was adopted, resulting in an actual power of 0.89 and minimum of four samples per group.^31^

**Figure 1:**
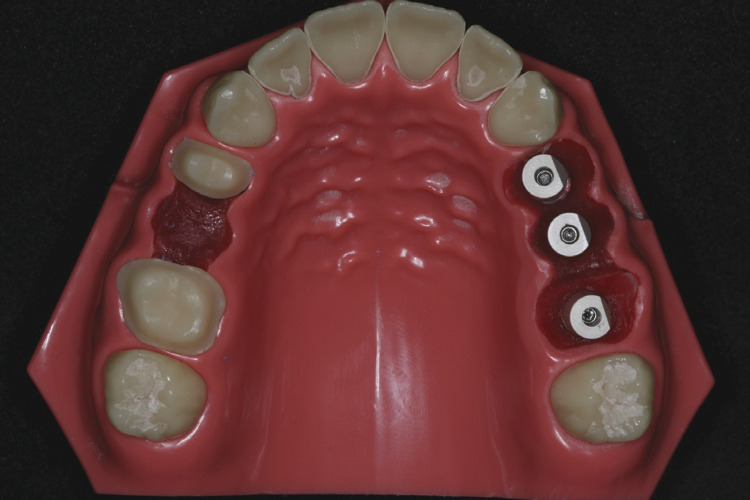
Maxillary typodont.

A typodont (P Oclusal, São Paulo, Brazil) with 3 digital analogs of Mini Abutment GM (Neodent, Curitiba, Brazil) for a fixed implant-supported prosthesis (EFF – dental components, São Paulo, Brazil) from the first maxillary left premolar to the first maxillary left molar was used as the master cast, simulating a partially edentulous maxillary posterior region (Figure 1). The control group, conventional chairside method (GC), and five fixed implant-supported temporary restorations were fabricated from the master cast using a conventional indirect provisional technique to produce the samples. Initially, compatible provisional titanium components (Mini Abutment GM, Neodent, Curitiba, Brazil) were adapted for the cast. They were then joined with acrylic resin (Vipicor, VIPI, Pirassununga, Brazil) and dental sculptures. For the GM and GP groups, scan bodies (Healing Scan, EFF Dental, São Paulo, Brazil) were installed in the master cast, five digital scans were installed in the master cast, and five digital scans were obtained using 3Shape TRIOS (3Shape, Copenhagen, Denmark). The .stl files were exported, and provisional restorations were designed (3Shape TRIOS /3Shape, Copenhagen, Denmark).

**Figure 2:**
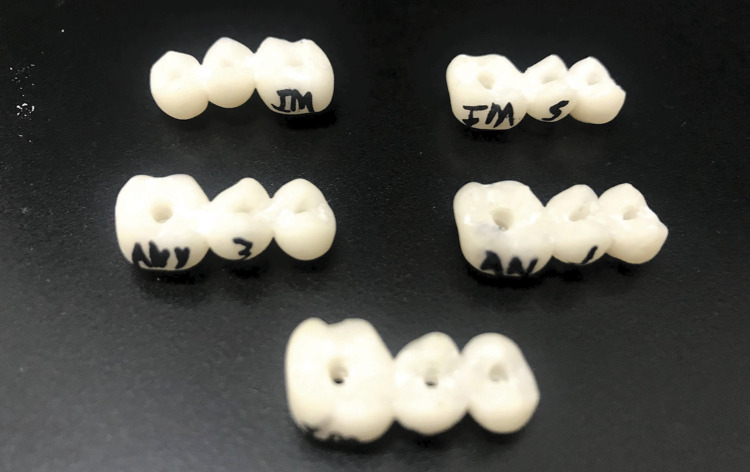
Specimens of all groups.

Five specimens were produced in each group (Figure 2) as follows: GM group: Telio CAD LT resin (Ivoclar Vivadent, Schaan, Liechtenstein) using a milling system (PrograMill PM7, Ivoclar, Schaan, Liechtenstein); GP group: Yller Cosmos Temp resin (Yller Biomaterials SA, Pelotas, Brazil; thickness, 50 μm) using a three-dimensional liquid crystal display (LCD) printer (Photon S; Anycubic, Shenzhen, China) following the guidelines in the printer according to the resin used; and GC group: chemical activate resin (Vipicor, VIPI, Pirassununga, Brazil), obtained by the conventional chairside method. A matrix was used to standardize all the specimens. The parameters used for printing were: thickness of each layer: 0.05 mm; exposure time: 12 min, cleaning: two baths of 5 min in isopropyl alcohol or ethanol; and post-cure time: up to 10 min in 72 watts UV chamber.

**Figure 3:**
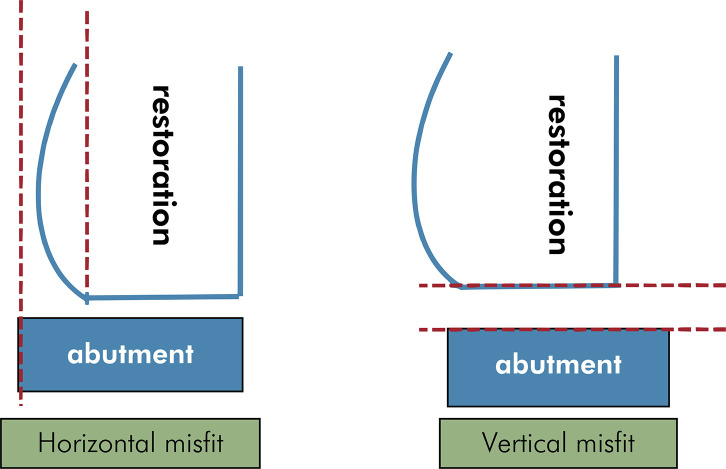
Schematic showing the vertical and horizontal misfit measurements.

**Figure 4:**
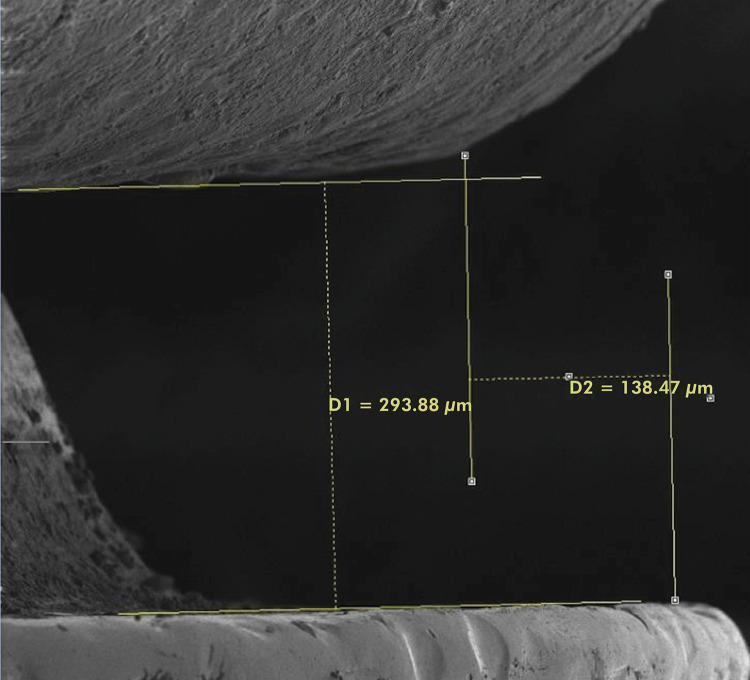
SEM image of the vertical and horizontal misfit.

The finishing and polishing processes were not performed on the specimens. The specimens were stored in a dry environment to protect them from external light exposure. Vertical and horizontal misfit^30^ (Figure 3) of the restoration interface were measured using SEM images under two conditions: one with only the central screw to stabilize the specimen and the other with all three screws tightened with 10 Ncm torque, as recommended by the manufacturer, using a prosthetic torque ranch (Neodent, Curitiba, Brazil). The mesial and distal gaps were analyzed for each temporary restoration, resulting in 15 measurements per group in each condition (one or three screws stabilizing the Sheffield Test),^25^ using the SEM Vega-specific analysis software (Figure 4).

Statistical analyses were performed using statistical software (R version 2.10.1; The R Foundation for Statistical Computing, Vienna, Austria) at a significance level of 5%. The data were checked for normality and homogeneity of variance. A logarithmic transformation was necessary for the data to satisfy the assumptions of the parametric analyses. Multiple comparisons were performed using the Tukey–Kramer test. The interfaces were evaluated in the vertical and horizontal directions with only the central screw, and all screws were tightened. In the horizontal evaluation, the measures were classified into three categories: overextension (over), underextension (under), and equal extension.^26^

## Results

**Figure 5:**
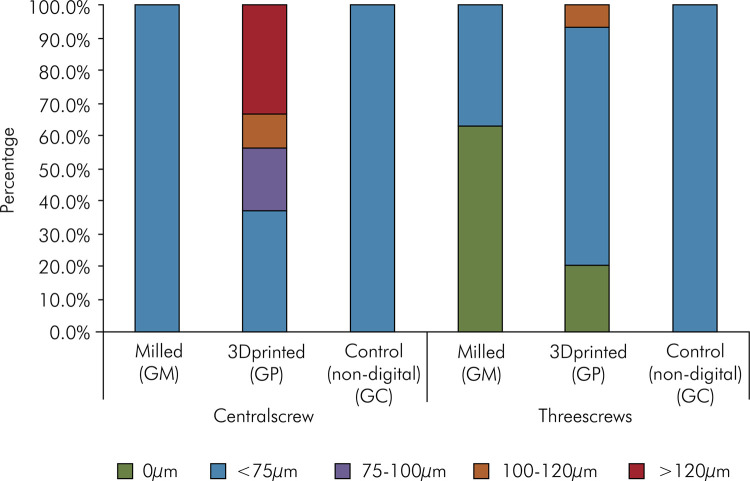
Vertical misfit of all tested groups.

In the evaluation using only the central screw or all screws, the GP group showed a significantly larger vertical misfit than the GM and GC groups (p < 0.0001) (Table 1). For restorations manufactured using all three techniques, the vertical misfit was significantly higher with one screw than with three screws (p = 0.0141) (Figure 5). Vertical misfit was segmented to better explain the data and interpret the differences between the production methods. All faces of both implants in the GM and GC groups showed a vertical misfit less than 75 μm in both situations. However, in the evaluation with only one central screw in the GP group, more than 60% of the implant faces presented with a misfit greater than 75 μm.

**Table 1:** Vertical misfit (μm) as a function of prosthesis production and evaluation.

Variable	Evaluation			

	Central screw		Three screws	

	Mean (SD)	Minimum/maximum value	Mean (SD)	Minimum/maximum value
(GM)	13.22 (7.64) Ab	7.46/26.43	1.3 (0.28) Bb	0.97/1.64
(GP)	110.41 (33.98) Aa	53.03/136.71	13.53 (14.83) Ba	3.96/39.83
(GC)	10.23 (2.34) Ab	6.46/12.38	4.12 (1.83) Bab	1.66/6.28

Central screw: only the central screw was tightened; three screws: all three screws were tightened. GM, milling method; GP, three-dimensional printing; GC, conventional chairside method. SD: standard deviation. Different letters (uppercase in horizontal and lowercase in vertical) indicate statistically significant differences (p < 0.05)

**Figure 6:**
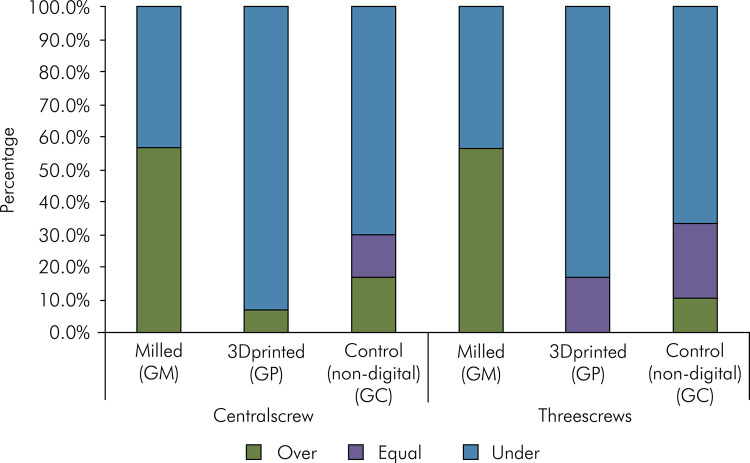
Horizontal misfit of all tested groups.

The GP group also showed greater horizontal misfit in the evaluation with only the central screw than with all three screws (p = 0.00068) (Table 2). In both situations, the horizontal misfit was significantly higher in the GP group than in the GM and GC groups (Figure 6).

**Table 2:** Horizontal misfit (μm) as a function of prosthesis production and evaluation.

Variable	Evaluation			

	Central screw		Three screws	

	Mean (SD)	Minimum/maximum value	Mean (SD)	Minimum/maximum value
(GM)	20.66 (4.22) Ab	(13.5/23.68)	20.37 (4.05) Ab	(13.62/23.68)
(GI)	122.81 (29.61) Aa	(71.96/147.64)	55.35(23.68) Ba	(28.36/84.59)
(GC)	20.97 (9.89) Ab	(9.32/32.87)	15.15 (6.05) Ab	(8.18/22.38)

Central screw: only the central screw was tightened; three screws: all three screws were tightened. GM, milling method; GP, three-dimensional printing; GC, conventional chairside method. Distinct letters (uppercase horizontally and lowercase vertically) indicate statistically significant differences (p < 0.05). p (prosthesis) < 0.0001; p (evaluation) = 0.0010; p (interaction) = 0.00068.

## Discussion

Based on the results of this study, both the null hypotheses were rejected. Under both conditions (*i.e.*, with only the central screw tightened and all three screws tightened), the GP group showed the highest vertical misfit. The results of this study also demonstrate that tightening all screws promotes better seating.

An important factor to emphasize in relation to the findings is that the classification of measures was performed to characterize the distribution of results and allow for a better assessment of the differences between manufacturing techniques in conditions with a single central screw or three screws. However, these gaps may enhance bacterial proliferation, which can lead to bone loss around the implant and generate malodor.^26^With only the central screw tightened, temporary restorations in all groups showed underextension. Since overextension is considered an unfavorable situation that causes difficulty in cleaning and could result in biological problems,^26^ these study findings indicate that these restorations can only be used for short periods of time.

Differences may exist between the tested materials, which could be a determining factor for the obtained results. The chemical composition (Table 3) can affect the properties of provisional crowns because they contain different chemical products and polymerization methods. For example, the printed group requires a post-curing method that requires careful handling. Analyzing these results consistently is crucial with regard to the chemical components of this resin and the polymerization method.

**Table 3:** Materials’ composition.

Material	Composition
Telio CAD Blocks Components (Ivoclar Vivadent)	Polymethyl methacrylate (PMMA), pigments. Telio stains components: bis-GMA, urethane dimethyl- acrylate and triethylene glycol dimethacrylate (86 wt. %), fillers and pigments (13 wt. %), catalysts, stabilizers
Yller Cosmos (Yller)	Oligomers, monomers, photoinitiators, stabilizers and pigment
Vipicor (VIPI)	Polymethylmethacrylate, benzoyl peroxide, pigments. Liquid- methyl methacrylate, EDMA (crosslink), inhibitor, fluorescent

Assessments of misfit in temporary restorations are extremely important, as misfit can cause mechanical or biological problems, compromising the longevity and success of the treatment.^26^The three-dimensionally printed restorations showed a greater discrepancy even in the horizontal evaluation. Reducing the size of the restoration may be beneficial for peri-implant health of the region, which is important for proper gingival conditioning. Restorations generated using these technologies should be used with caution and only for short periods, from 15 days to 6 months, until installation of the definitive prosthesis. Clinical adjustments must be made to guarantee minimal misfit.

Regarding the materialization of definitive crowns, in most cases, subtractive techniques that present better marginal adaptation than additive 3D printing techniques are used.^4,9^ However, the use of additive techniques for the materialization of implant-supported rehabilitation has been increasing because they present less material waste, presenting itself as a more sustainable technique associated with a more accessible cost value compared to milling machines for machining. In addition, digital flow is an important tool for fabricating prostheses at high speed.

Manufacturing methods based on milling and three-dimensional printing are being studied in dentistry because of their high applicability.^7,9,10^Three-dimensionally printed restorations show more discrepancies in both vertical and horizontal evaluations. The accuracy of AM methods may be influenced by the material utilized and post-processing procedures.^9-12^ The resin used for printing temporary restorations was compatible with three-dimensional LCD printers based on stereolithography (SLA) technology, and the post-polymerization process was in accordance with the resin manufacturer’s guidelines. Future studies should compare the performance of the same resin using different printers to assess the performance of the material. In addition, restorations obtained using these printers can be used for short periods without causing further clinical damage. Clinical adjustments must be made to guarantee minimal misfit.

Various approaches have been used to assess the marginal fit, including stereomicroscopy, scanning electron microscopy, optical microscopy, and microcomputed tomography (m-CT). Employing stereomicroscopic techniques requires a transverse section of both the crown and tooth to quantify misfit; however, this procedure has the potential to induce deformations. Employment of the mCT system presents a relatively costly yet non-destructive avenue for appraising marginal fit. This three-dimensional, high-resolution imaging system provides intricate cross-sectional insights into crown-to-die fit while safeguarding the integrity of the specimen. Investigations involving SEM require the specimen to be appropriately aligned to perform accurate measurements.

Changes in the parameters resulted in differences in the build and machining times, which could have contributed to changes in the post-polymerization period.^18^ The increased use of three-dimensional printing (SLA) represents significant progress in digital workflow. Further studies are needed to evaluate and compare the materials used with different technologies in three-dimensional printers.

## Conclusion

Restorations prepared using the conventional chairside method and milling showed more favorable results than three-dimensionally printed restorations. The methods and materials used to manufacture temporary restorations can influence their characteristics in relation to marginal fit.
